# Key points in writing instrument development and psychometric evaluation reports

**DOI:** 10.1590/0034-7167-2025-0006

**Published:** 2025-10-03

**Authors:** Maryam Alizadeh, Hooman Shahsavari, Pegah Matourypour

**Affiliations:** I Tehran University of Medical Sciences (TUMS), School of Medicine and Health Professions Education Research Center, Department of Medical Education. Tehran, Iran; II Tehran University of Medical Sciences, Nursing and Midwifery Faculty, Medical Surgical Nursing Department. Tehran, Iran

Dear Dr Dulce Aparecida Barbosa

Editor in Chief of the Revista Brasileira de Enfermagem

In the articles related to the instrument development and psychometric evaluation published in various reputable and international journals, a noteworthy point frequently seen is that, some articles have comprehensively described all stages of instrument development and its psychometric evaluation, Meanwhile, some of them have only addressed some aspects of instrument development, or explained just some stages of development and introduced the designed instrument in its final form. In this regard, important question that arises is that how much valid and reliable these instruments actually are. In the other hand some instrument development points, such as the research method, have been reported in different ways in these type of articles. Sometimes, no information has been provided regarding the scoring system or the time required to complete the designed instrument, which causes some confusions for the readers or researchers who want to use the instrument.

In this letter, we intend to state the key and practical points in each phase. Also, we will point out the minimum standard explanation that should be provided by the instrument development studies.

The method that used in instrument development studies is reported variable in different articles, some articles report as descriptive, analytic as method, which are not acceptable, however instrument development references mention to method as methodological^(1,2)^. Mixed method studies can also be used when a qualitative approach is used in the item production stage to produce items, interviews and qualitative analysis are conducted, then the questionnaire is completed in testing stage and exploratory factor analysis is performed followed by quantitative analysis^(2,3)^.

Also scoring of finalized developed instrument in some of instrument development articles was not reported, however it is so important for using other researchers and who interest to use developed instrument, so it should be reported. At the end of the research, the developed instrument should be introduced in terms of the number of items and dimensions. It is suggested that in this section, in order to complete the instrument introduction, the time required to complete the instrument should also be mentioned^(4)^.

The instrument’s scoring system should also be mentioned (Likert scale, yes-no, Multiple choice Question (MCQ), etc.). The Likert scale is usually appropriate for self-report tools. Reversed items, scoring system, cutoff point and division degree of variables should also be reported^(1)^.

Reference texts on the instrument development and psychometric evaluation introduce the following steps that should be considered in this regard: 1) Items generation, 2) initial construction of instrument and validity assessment, 3) Field testing, and 4) Factor analysis and reliability assessment. They also suggest all these steps to be carried out and completed in the instrument development studies^(1,5)^. The important point in observing these steps is that, in addition to assess face and content validity, construct validity is achieved through factor analysis and highly recommended. By examining construct validity in this way, convergent and divergent validity, which is inherent in factor analysis, is also achieved^(2,6)^.

A point worth considering in this regard is to determine whether confirmatory factor analysis should also be performed or not. In fact, this point is challenging for many researchers who try to develop instruments.

the confirmatory type is the test hypothesis, suggesting that whether the statements included within each dimension are really fit and belong to that dimension or not^(7)^. The exploratory type, as the name implies, involves the detection and determining the dimensions. CFA is preferable to EFA as an approach to construct validity because CFA is a hypothesis testing approach-testing the hypothesis that the items belong to specific factors, rather than having the dimensionality of a set of items emerge empirically, as in EFA^(1)^.

However, experts in instrument development recommend that after conducting exploratory factor analysis to test the hypothesis and ensure the dimensions, it is better to confirm them by conducting confirmatory factor analysis^(1)^. In other words, exploratory factor analysis is essential, but confirmatory factor analysis is optional in instrument development studies.

We intend to express some key points in each stage of instrument development:

## Item generation:

This stage can be done with deductive, inductive or both approaches depending on the nature of concept under study, the researcher’s time and the amount of texts and evidence available. In the deductive approach, the theoretical foundations of concept have sufficient information on how to develop the instrument’s items, and the researcher must gain a proper understanding of the phenomenon under study. Therefore, the researcher reviews various evidence in national and international scientific databases and also in grey literature, and then extracts appropriate items by searching for keywords related to the concept under study in order to form pool of items. If there are not many texts available, the researcher can use the inductive approach. In this way, qualitative interviews are conducted and data analysis is performed to form the instrument’s items. At this stage, no judgment is made about the suitability of extracted items, and the goal is to form a large pool of items^(5)^. If there is sufficient evidence on the concept under study, it is recommended to use both deductive and inductive approaches^(2,4)^. This ensures that the necessary scientific foundations have been taken into account in the production of items and they have been formed in accordance with the culture, beliefs, and values of the society in which the instrument is to be used^(3)^. Walker and Avant suggest concept analysis can be performed in developing pool of items^(8)^.

## Initial construction of instrument and its validity assessment:

In this stage, the codes extracted in the previous stage are listed and compiled to form the initial tool. The highest possible number of items is then included in the process at this stage to prevent the information loss (pool of items)^(4)^. Initial review of the items is carried out in a meeting (research team) to initially correct the sentences and eliminate obvious overlaps of the items^(7)^. Then, face and content validity is carried out by a panel of experts, and CVI, CVR and Kappa coefficient are reported^(1,6)^ to find out whether items fully evaluate the desired concept. This is accomplished with exploratory factor analysis. Exploratory factor analysis uses factor analysis to first determine the construct validity and then, reduce the items and dimensions of developed instrument, which is consistently mentioned in the reference texts^(1,2,5,7)^. On the other hand, by conducting exploratory factor analysis, convergent and divergent validity is also achieved. In this way, items that are placed in one dimension have convergent validity, and their semantic difference with another dimension shows divergent validity^(2,6)^. Factor analysis also classifies the instrument’s dimensions by eliminating irrelevant items. It is suggested to perform exploratory factor analysis in all instrument development studies using SPSS software.

## Field testing:

Honking considers the appropriate sample size for field testing to be 4 to 10 times of the items^(5)^. Polit considers the sample size required for factor analysis to be 300 samples^(1)^. Others have considered the minimum sample size to be at least 150 people for exploratory factor analysis and 200 for confirmatory factor analysis^(5)^. After determining the sample size, the researcher should use Bartlett’s factor analysis to ensure the adequacy of sampling and the ability to perform factor analysis (KMO), taking into account the data obtained from construct validity^(1,5,7)^. The reliability of designed instrument can also be carried out with different methods using the resulting data. Reliability can also be examined with different methods, one of the most common of which is to assess internal correlation through Cronbach’s alpha^(1,9)^.

The purpose of mentioning the above information is to help clarify important points that should be considered in the design and psychometric evaluation of instruments.
